# Modulation of human endogenous retroviruses and cytokines expression in peripheral blood mononuclear cells from autistic children and their parents

**DOI:** 10.1186/s12977-022-00603-6

**Published:** 2022-11-30

**Authors:** Chiara Cipriani, Martina Giudice, Vita Petrone, Marialaura Fanelli, Antonella Minutolo, Martino T. Miele, Nicola Toschi, Christian Maracchioni, Martina Siracusano, Arianna Benvenuto, Antonella Coniglio, Paolo Curatolo, Luigi Mazzone, Grelli Sandro, Enrico Garaci, Paola Sinibaldi-Vallebona, Claudia Matteucci, Emanuela Balestrieri

**Affiliations:** 1 Department of Experimental Medicine, University of Rome Tor Vergata, Via Montpellier 1, 00133 Rome, Italy; 2 Department of Biomedicine and Prevention, Tor Vergata University of Rome, 00133 Rome, Italy; 3 Martinos Center for Biomedical Imaging and Harvard Medical School, Boston, USA; 4 Child Neurology and Psychiatry Unit, System Medicine Department, Tor Vergata University Hospital of Rome, 00133 Rome, Italy; 5 Virology Unit, Policlinic of Tor Vergata, 00133 Rome, Italy; 6 University San Raffaele, Rome, Italy; 7 IRCCS San Raffaele Pisana, 00133 Rome, Italy; 8 Institute of Translational Pharmacology, National Research Council, 00133 Rome, Italy

**Keywords:** HERVs, HEMO, Cytokines, Gene expression, In vitro treatment, Antiretrovirals, Biomarker, Mother–child association, Autism spectrum disorder

## Abstract

**Background:**

Putative pathogenic effects mediated by human endogenous retroviruses (HERVs) in neurological and psychiatric disorders in humans have been extensively described. HERVs may alter the development of the brain by means of several mechanisms, including modulation of gene expression, alteration of DNA stability, and activation of immune system. We recently demonstrated that autistic children and their mothers share high expression levels of some HERVs and cytokines in peripheral blood mononuclear cells (PBMCs) ex vivo, suggesting a close mother–child association in Autism Spectrum Disorder (ASD).

**Results:**

In the present study, PBMCs from autistic children and their parents were exposed to stimulating factors (Interleukin-2/Phytohaemagglutinin) or drugs, as Valproic acid and Efavirenz. The results show that HERVs and cytokines expression can be modulated in vitro by different stimuli in PBMCs from autistic children and their mothers, while no significant changes were found in PBMCs ASD fathers or in controls individuals. In particular, in vitro exposure to interleukin-2/Phytohaemagglutinin or valproic acid induces the expression of several HERVs and cytokines while Efavirenz inhibits them.

**Conclusion:**

Herein we show that autistic children and their mothers share an intrinsic responsiveness to in vitro microenvironmental changes in expressing HERVs and pro-inflammatory cytokines. Remarkably, the antiretroviral drug Efavirenz restores the expression of specific HERV families to values similar to those of the controls, also reducing the expression of proinflammatory cytokines but keeping the regulatory ones high. Our findings open new perspectives to study the role of HERVs in the biological mechanisms underlying Autism.

**Supplementary Information:**

The online version contains supplementary material available at 10.1186/s12977-022-00603-6.

## Introduction

Autism Spectrum Disorder (ASD) is a complex and heterogeneous neurodevelopmental condition affecting more than 1% of children [1]. ASD diagnosis is based on clinical observations and neuropsychological assessment of the early-onset dysfunctions in social-communicative reciprocity and restricted and repetitive patterns of behaviour, interests, or activities [2]. ASD is believed to have a multifactorial and complex aetiology, mainly attributed to the combination of genetic vulnerability and environmental factors [3]. Numerous ASD-risk genes, chromosomal abnormalities, polymorphisms, copy number variation and de novo mutations have been identified in the ASD pathogenesis [4, 5], nevertheless, genetics alone cannot explain the heterogeneity of clinical phenotypes and the concordance rate described in mono- and di-zygotic twin pairs [6, 7].

The involvement of the gut-brain axis in the etiology of autism has also been proposed. Studies on maternal immune activation and interventions on gut microbiota dysbiosis in animal model, seem to support this hypothesis. Lipopolysaccharide-mediated maternal immune activation induces an abnormal brain-gut-microbiota axis with social behavior deficits, anxiety-like and repetitive behavior, hypomyelination, and an ASD-like microbiota profile in offspring [8]. Moreover, fecal transplant from healthy donor animals, modulates the gut microbiota components and rescues social impairment in a rat model of autism, suggesting this approach as possible intervention in a preclinical setting [9].

Over the recent years, the research efforts focused on the understanding of the biological mechanisms that guide the pathophysiology of ASD and it is now established that environmental factors (e.g. toxicants, maternal intake of medication and maternal infections) can act mostly between conception and birth, in specific well-delineated sensitive time-windows of increased vulnerability of the nervous system [10]. Indeed, during pregnancy, the maternal immune response induced by several environmental insults could affect different stages of neurodevelopment in the foetus, contributing to the appearance of altered phenotypes early in childhood as well as adult or aged progeny, highlighting the close interconnection between the immune and the nervous system [11].

In this complex scenario, Human Endogenous Retroviruses (HERVs) have been proposed as contributing factors involved in autism, spanning the bridge between genetic susceptibility, environmental risk factors and immune response [12].

HERVs are genetic elements, derived from their exogenous retroviral counterpart by a process of germline infection and proliferation within the human genome [13–15], and their integration as proviruses led to the fixation and the vertical transmission, following Mendelian laws [16]. HERVs currently make up ~ 8% of the genetic material [17, 18], resulting from the proliferation of a few initial germline invasions by exogenous retroviruses [19, 20]. As a consequence, extensive interindividual variability due to new insertions, copy number variations, unfixed copies, and polymorphisms has been demonstrated [21–23]. During co-evolution with humans, the majority of HERV sequences were silenced by negative selective pressure and/or mutations [18], and, conversely, some of them have been coopted for physiological functions [24]. Their activation in response to external stimuli has been also associated with human pathological conditions. Indeed, growing evidences highlight the involvement of HERV-derived transcripts in complex diseases including various cancer types, autoimmune, neurological and psychiatric disorders such as schizophrenia, attention deficit/ hyperactivity disorder (ADHD) and also ASD [12, 25–28].

In previous works, we highlighted the presence of a distinct expression profile of some HERV families (HERV-H, HERV-K, and HERV-W) in fresh PBMCs from ASD and ADHD children [30]. Particularly, high levels of HERV-H were found both in ASD and ADHD children compared to typical developing individuals and closely related with more severe clinical phenotype, being more expressed in children with severe motor and communication impairment [29, 30]. Moreover, in a recent study including ASD children and their parents, we demonstrate that autistic children and their mothers share high expression levels of HERV-H and HEMO [an envelope gene, named human endogenous MER34 (medium-reiteration-frequency-family-34) ORF] [31] and cytokines as tumor necrosis factor alpha (TNF-α), interferon gamma (IFN-γ) and IL-10, suggesting a close mother–child association in ASD [32].

This observation is corroborated by the results obtained in two preclinical models (inbred BTBR T + tf/J and valproate-treated CD1 mice) in which high expression levels of ERVs and proinflammatory cytokines and Toll-Like Receptors were associated with ASD-like phenotype [33]. Interestingly, in the valproate-treated mice, the increased expression of ERVs and behavioural alterations were inherited across generations via maternal lineage [34], further confirming a pivotal role of the maternal molecular profile in the acquisition of autistic phenotype.

Based on our previous findings, in the present study, peripheral blood mononuclear cells from autistic children and their parents were cultured in the presence of the T cell-stimulating factor Interleukine-2 and the mitogen phytohemagglutinin or drugs such as the histone deacetylase inhibitor valproic acid and the non-nucleoside reverse transcriptase inhibitor efavirenz (EFV), with the intent to investigate whether the expression level of HERVs and cytokines could be modulated by exposure to different stimuli or drugs.

## Results

The stimulation in culture with interleukin-2 and phytohaemagglutinin modulated the expression of HERVs and cytokines in PBMCs from autistic children and their mothers.

**Fig. 1:**
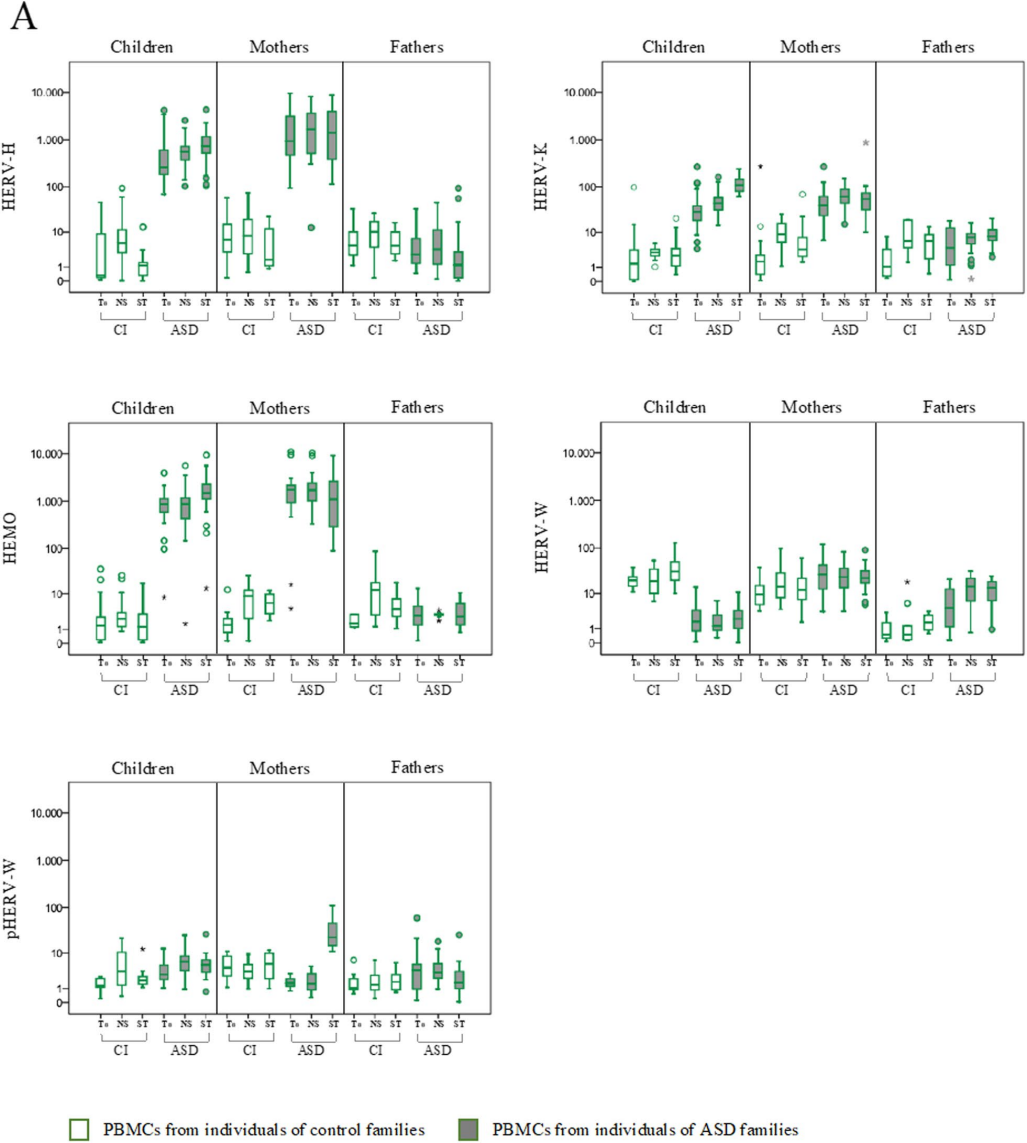

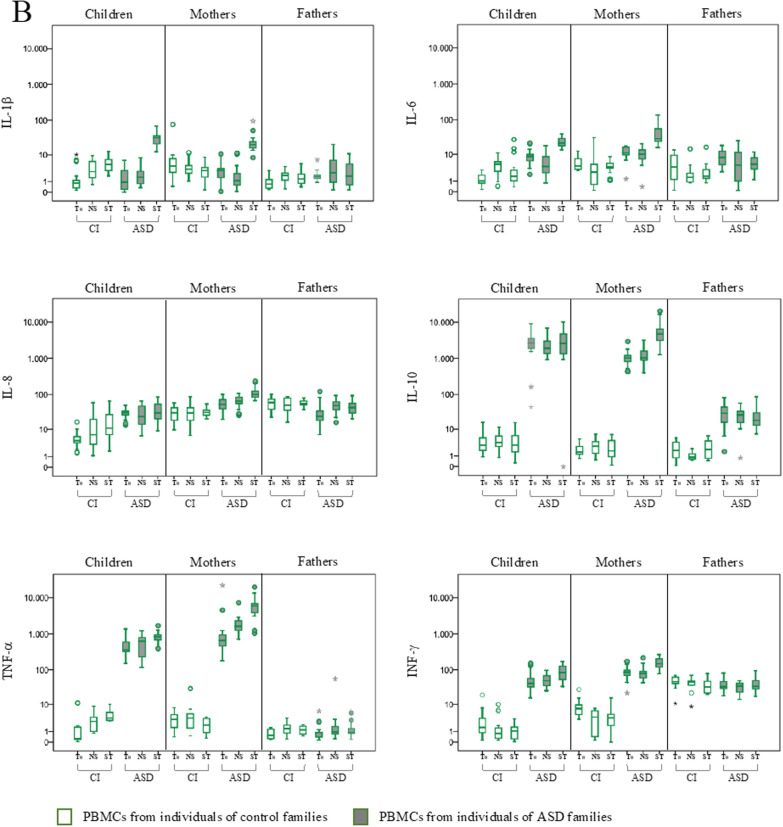
HERVs and cytokines expression in PBMCs from ASD children, their parents and control individuals in different culture conditions. HERV transcriptional activity (**A**) and cytokines expression levels (**B**) were evaluated at baseline (T_0_), after culture without any stimulation (NS) and in presence of IL-2 and PHA (ST) by RT-Real time PCR. The results are represented as box plots, depicting mild (black dot) and extreme outliers (asterisk) for each group. Green bordered white boxes = control individuals; Green bordered grey boxes = ASD children and their parents

The expression of HERV-H, HERV-K, HERV-W and pHERV-W env gene, HEMO and a selected group of cytokines (IL-1β, IL-6 IL-8, IL-10, TNF-α, and IFN-γ) was evaluated in PBMCs from ASD children and their parents and from corresponding control individuals (CI), by quantitative RT-Real time PCR analysis. All samples were analyzed at the sampling time (baseline, T_0_) and after 72 h in culture without any stimulation (NS) or in presence of Interleukin-2 (IL-2) and phytohaemagglutinin (PHA) (ST). The results are represented as box plots in Fig. 1 and statically significant comparisons are listed in Table 1. Taking into account the interindividual variations already present at baseline in all groups of individuals analyzed, we chose to consider only gene expression variations for 2^−∆∆Ct^ > 10 [29] (see Additional file 1: Tables S1 for median values and interquartile range, IQR).

**Table 1:** Non-parametric Friedman test on HERV and cytokines expression in PBMCs stimulated in vitro with IL-2/PHA

		HERV-H	HERV-K	HEMO	HERV-W	pHERV-W	IL-1β	IL-6	IL-8	IL-10	TNF-α	INF-γ
Children	T_0_* vs* NS	0.001	–	–	–	0.001	0.001	0.005	–	–	0.011	–
CI	T_0_ *vs* ST	–	–	–	0.004	–	< 0.001	–	–	–	< 0.001	–
	NS *vs* ST	–	–	–	0.001	–	–	–	–	–	–	–
Children	T_0_* vs* NS	–	–	–	–	–	–	–	–	–	–	–
ASD	T_0_ *vs* ST	0.002	< 0.001	0.002	–	–	< 0.001	< 0.001	–	–	< 0.001	< 0.001
	NS *vs* ST	–	< 0.001	0.003	–	–	< 0.001	< 0.001	–	–	0.001	0.002
Mothers	T_0_* vs* NS	–	0.003	0.004	–	–	–	–	–	–	–	–
CI	T_0_* vs* ST	–	–	0.006	–	–	–	–	–	–	–	–
	NS *vs* ST	–	–	–	–	–	–	–	–	-	–	–
Mothers	T_0_* vs* NS	–	0.012	–	–	–	–	–	–	–	0.003	–
ASD	T_0_* vs* ST	–	–	–	–	< 0.001	< 0.001	< 0.001	< 0.001	< 0.001	< 0.001	< 0.001
	NS *vs* ST	–	–	–	–	< 0.001	< 0.001	< 0.001	< 0.001	< 0.001	0.001	< 0.001
Fathers	T_0_* vs* NS	–	–	–	–	–	–	–	–	–	-	–
CI	T_0_ *vs* ST	–	–	–	–	–	–	–	–	–	-	–
	NS *vs* ST	–	–	–	–	–	–	–	–	–	-	–
Fathers	T_0_ *vs* NS	–	–	–	< 0.001	–	–	–	–	–	-	–
ASD	T_0_ *vs *ST	–	–	–	0.006	–	–	–	–	–	-	–
	NS *vs* ST	0.020	–	–	–	0.008	–	–	–	–	-	–

*HERVs* human endogenous retroviruses, *PBMCs* peripheral blood mononuclear cells, IL-: Interleukin, *TNF* tumor necrosis factor, *IN*F interferon, *CI* control individuals, *ASD* autism spectrum disorder, *T0*: basaline, *NS* not stimulated, *S*T stimulated, *VPA* Valproic acid, *EFV* efavirenz

In PBMCs from ASD children stimulation in culture with IL-2 and PHA significantly increased the expression of several HERVs (panel A) and cytokines (panel B) compared with both the not stimulated condition (HERV-K, HEMO, IL-1β, IL-6, TNF-α, IFN-γ) and the baseline (T_0_) (HERV-H, HERV-K, HEMO, IL-1β, IL-6, TNF-α, IFN-γ).

In ASD mothers the stimulation significantly increased only pHERV-W and all the cytokines analyzed in comparison to NS and T_0_ condition (IL-1β, IL-6, IL-8, IL-10, TNF-α, INF-γ).

In PBMCs from ASD fathers kept in culture, significantly higher levels of HERV-W respect to T_0_ were always observed regardless of the presence of IL-2 and PHA in the culture medium.

Concerning control individuals, the stimulation with IL-2 and PHA significantly increased the expression of HERV-W in PBMCs from children, respect to both the NS and T_0_ conditions. All other gene expression changes observed in control individuals, although statistically significant, did not reach values of 2^−∆∆Ct^ > 10.

Finally, at the baseline, HERVs and cytokines expression levels observed in ASD children and their parents were in line with what we already described [32]. Specifically, ASD children and their mothers showed significantly higher expression levels of HERV-H, HERV-K and HEMO respect to controls, while in ASD fathers only HERV-W was significantly higher respect to corresponding control groups. Of note, here we describe for the first time that the expression levels at T_0_ of pHERV-W were significantly higher in ASD children and lower in their mothers, respect to matched controls. Regarding the expression of the cytokines in PBMCs from ASD children and their mothers IL-6, IL-8, IL-10, TNF-α and INF-γ was significantly higher compared to corresponding controls.

Moreover, in order to compare stimulation response across groups (ASD and CI) while taking confounds into account, for each stimulus we employed linear mixed models, considering each biomarker as the dependent variable and including timepoint, as repeated within-subject factor (baseline vs 72 h), a between-subject factor (diagnostic group), and a timepoint*diagnostic group interaction term. In children, for the stimulus Il2/PHA we found a statistically significant timepoint*diagnostic group interaction effect on the induction of HERV-K (p < 0.001), HEMO (p < 0.001), HERV-W (p = 0.001), IL-1 → (p < 0.001), IL-6 (p < 0.011), TNF-α (p < 0.001) and INF-γ (p = 0.017), as well as in mothers on the induction of pHERV-W (p < 0.001) and all cytokines considered (IL-1β p < 0.001; IL-6 p < 0.001; IL-8 p = 0.001; IL-10 p < 0.001; TNF-α p = 0.007; INF-γ p < 0.001), with the largest effect in ASD group as compared to the control group. In PBMCs kept in culture without any stimulation, no evidence of interaction effect in children and their mothers and fathers have been found.

### *The *in vitro* treatment with valproic acid induced HERV and cytokines expression in PBMCs from ASD children and their mothers*

**Fig. 2:**
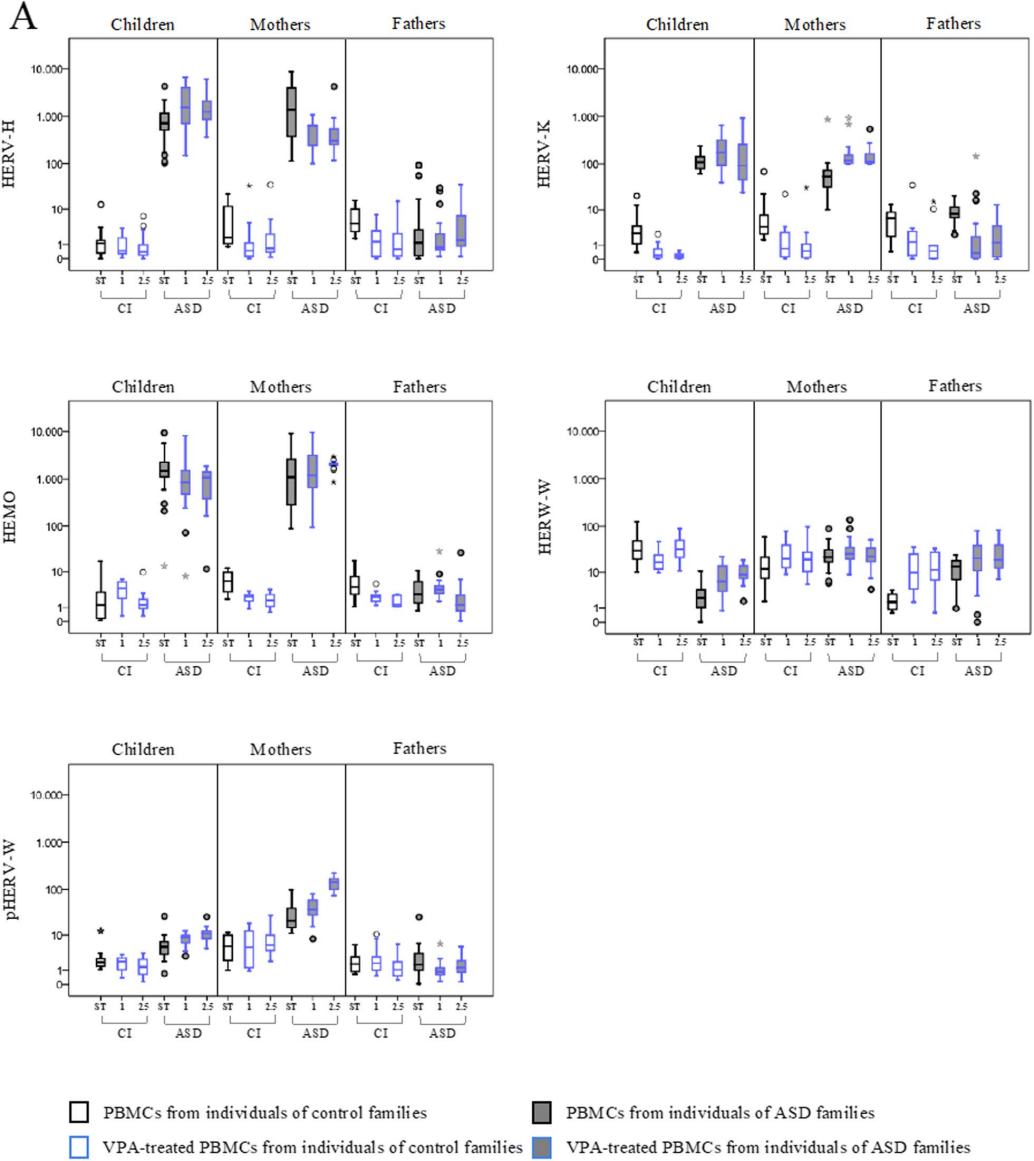

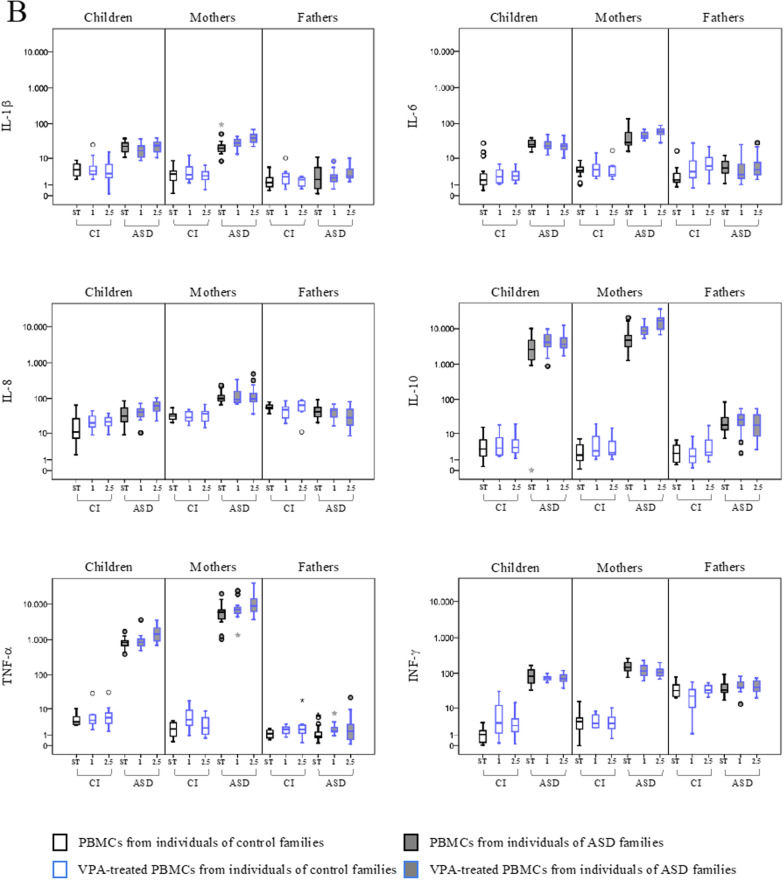
HERVs and cytokines expression in PBMCs from ASD children, their parents and control individuals treated with VPA. HERV transcriptional activity (**A**) and cytokines expression levels (**B**) were analysed in PBMCs treated with Valproic acid (VPA) at a final concentration of 2.5 µM (2.5) and 1 µM (1)) by RT-Real time PCR. The results are represented as box plots, depicting mild (black dot), and extreme outliers (asterisk) for each group. Blue bordered white boxes = control individuals; Blue bordered grey boxes = ASD children and their parents

PBMCs from ASD children and their parents and corresponding controls were exposed in vitro to valproic acid (VPA) treatment at the final concentration of 1 and 2.5 µM, and the expression of HERVs and cytokines was evaluated (Fig. 2, Table 2). Median values and IQR of the experimental conditions are reported in Additional file 1: Table S1.

**Table 2:** Non-parametric Friedman test on HERV and cytokines expression in PBMCs exposed in vitro to VPA

		HERV-H	HERV-K	HEMO	HERV-W	pHERV-W	IL-1β	IL-6	IL-8	IL-10	TNF-α	INF-γ
Children	ST *vs *VPA 1 μM	-	0.001	0.016	0.031	-	-	-	-	-	-	0.011
CI	ST *vs *VPA 2.5 μM	-	< 0.001	-	-	-	-	-	-	-	-	0.024
	VPA 1 μM *vs* VPA 2.5 μM	-	-	0.020	0.011	-	-	-	-	-	-	-
Children	ST *vs *VPA 1 μM	0.036	-	0.031	0.010	0.008	0.029	-	-	-	-	-
ASD	ST *vs *VPA 2.5 μM	0.027	-	0.036	< 0.001	< 0.001	0.040	-	0.001	-	<0.001	-
	VPA 1 μM *vs* VPA 2.5 μM	-	-	-	-	-	-	-	-	-	0.024	-
Mothers	ST *vs *VPA 1 μM	0.018	0.001	0.002	-	-	-	-	-	-	-	-
CI	ST *vs *VPA 2.5 μM	-	< 0.001	< 0.001	-	-	-	-	-	-	-	-
	VPA 1 μM *vs* VPA 2.5 μM	-	-	-	-	-	-	-	-	-	-	-
Mothers	ST *vs *VPA 1 μM	< 0.001	< 0.001	-	-	-	-	-	-	< 0.001	-	-
ASD	ST *vs *VPA 2.5 μM	< 0.001	< 0.001	-	-	< 0.001	<0.001	0.001	-	< 0.001	0.001	-
	VPA 1μM *vs* VPA 2.5 μM	-	-	-	-	< 0.001	0.018	-	-	-	-	-
Fathers	ST *vs *VPA 1 μM	-	-	-	0.004	-	-	-	-	-	-	-
CI	ST *vs *VPA 2.5 μM	0.028	0.021	< 0.001	0.002	-	-	0.027	-	-	-	-
	VPA 1mM *vs* VPA 2.5 μM	-	-	-	-	-	-	-	-	-	-	-
Fathers	ST vs VPA 1 μM	-	< 0.001	-	< 0.001	-	-	-	0.039	-	-	-
ASD	ST *vs *VPA 2.5 μM	-	< 0.001	-	0.041	-	-	-	0.040	-	-	-
	VPA 1 μM *vs* VPA2.5 μM	-	-	-	-	-	-	-	-	-	-	-

*HERVs* human endogenous retroviruses, *PBMCs* peripheral blood mononuclear cells, *IL*- Interleukin, *TNF* tumor necrosis factor, *INF*, interferon, *CI* control individuals, *ASD*, autism spectrum disorder, *T0* basaline, *NS* not stimulated, *ST* stimulated, *VPA* Valproic acid, *EFV*, efavirenz

In PBMCs from autistic children, the exposure to VPA (at both concentrations) significantly induced the expression of HERV-H, HERV-W and pHERV-W (panel A). Concerning cytokines expression, VPA modulated the expression of IL-8 and TNF-α at the higher concentration (panel B) compared to the untreated condition (Table 2).

In PBMCs from ASD mothers, VPA treatment also modulated HERV and cytokines expression. Specifically, VPA significantly increased HERV-K (at both concentrations), and pHERV-W (VPA 2.5 µM), and reduced HERV-H expression at both concentrations. VPA treatment also induced IL-1β, IL-6 and TNF-α at the concentration 2.5 µM, while IL-10 was induced at both concentrations, in comparison to untreated PBMCs.

In father groups (CI and ASD), VPA treatment at both concentrations significantly induced the expression of HERV-W only. All other gene expression changes observed in control individuals, although statistically significant, did not reach values of 2^−∆∆Ct^ > 10.

Moreover, as for the stimulus IL2/PHA, the linear mixed models employed for VPA at both concentrations analysed led to a statistically significant stimulus*diagnostic group interaction effect in children on the induction of HERV-H (p ≤ 0.004), HERV-K (p ≤ 0.031), pHERV-W (p < 0.001), IL-1 → (p ≤ 0.001), IL-6 (p < 0.001), TNF-α (p ≤ 0.017) and INF-γ (p ≤ 0.050) and in mother on the induction of HERV-K (p ≤ 0.030), pHERV-W (p < 0.001), IL-1 → (p < 0.001), IL-6 (p < 0.001), IL-8 (p ≤ 0.011) IL-10 (p < 0.001), TNF-α (p ≤ 0.026) and INF-γ (p ≤ 0.022), with the largest effect in ASD group as compared to the control group, while no statically significant results were found in fathers.

### The in vitro treatment with Efavirenz reduces HERV and cytokines expression in PBMCs from ASD children and their mothers

**Fig. 3:**
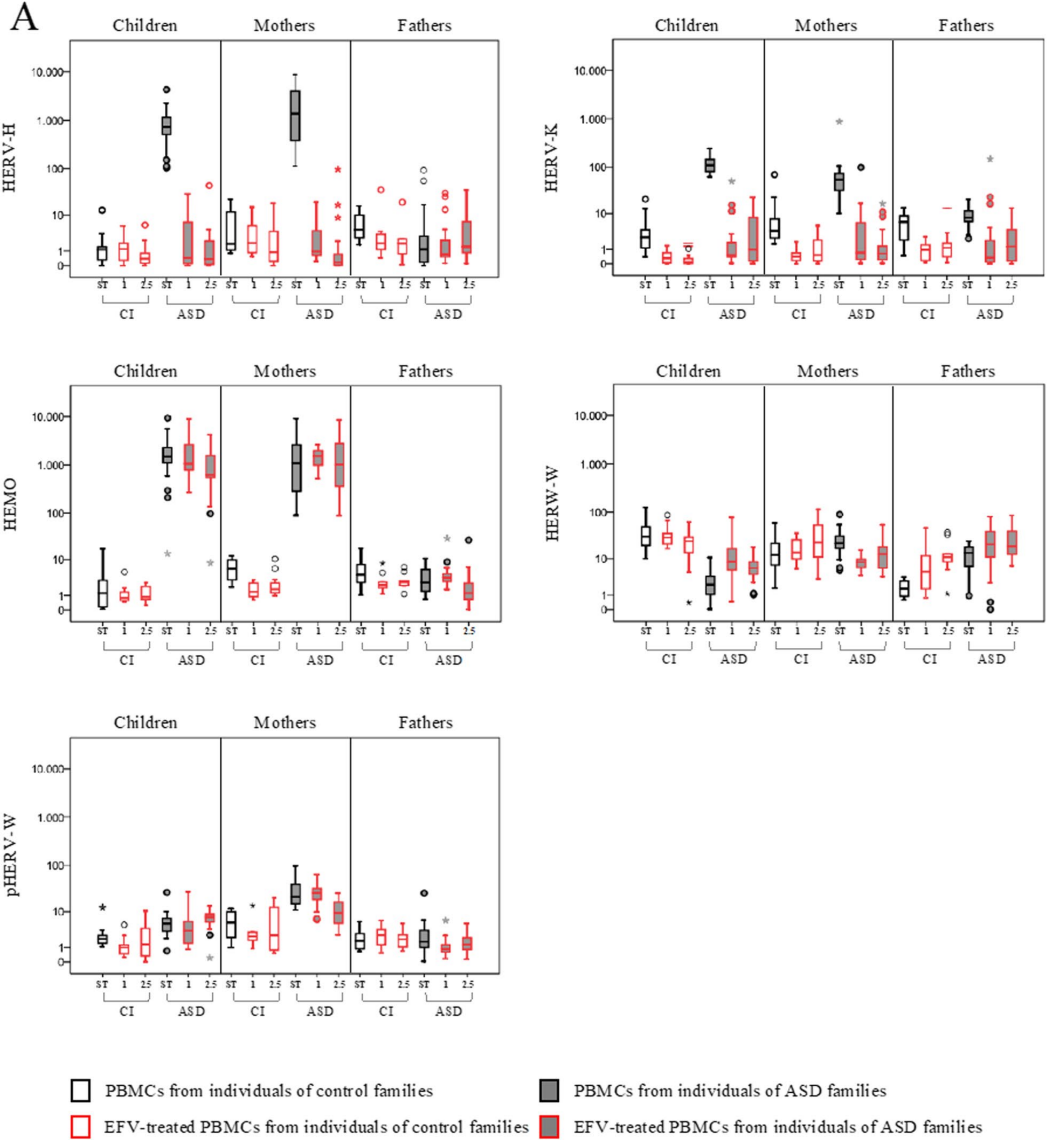

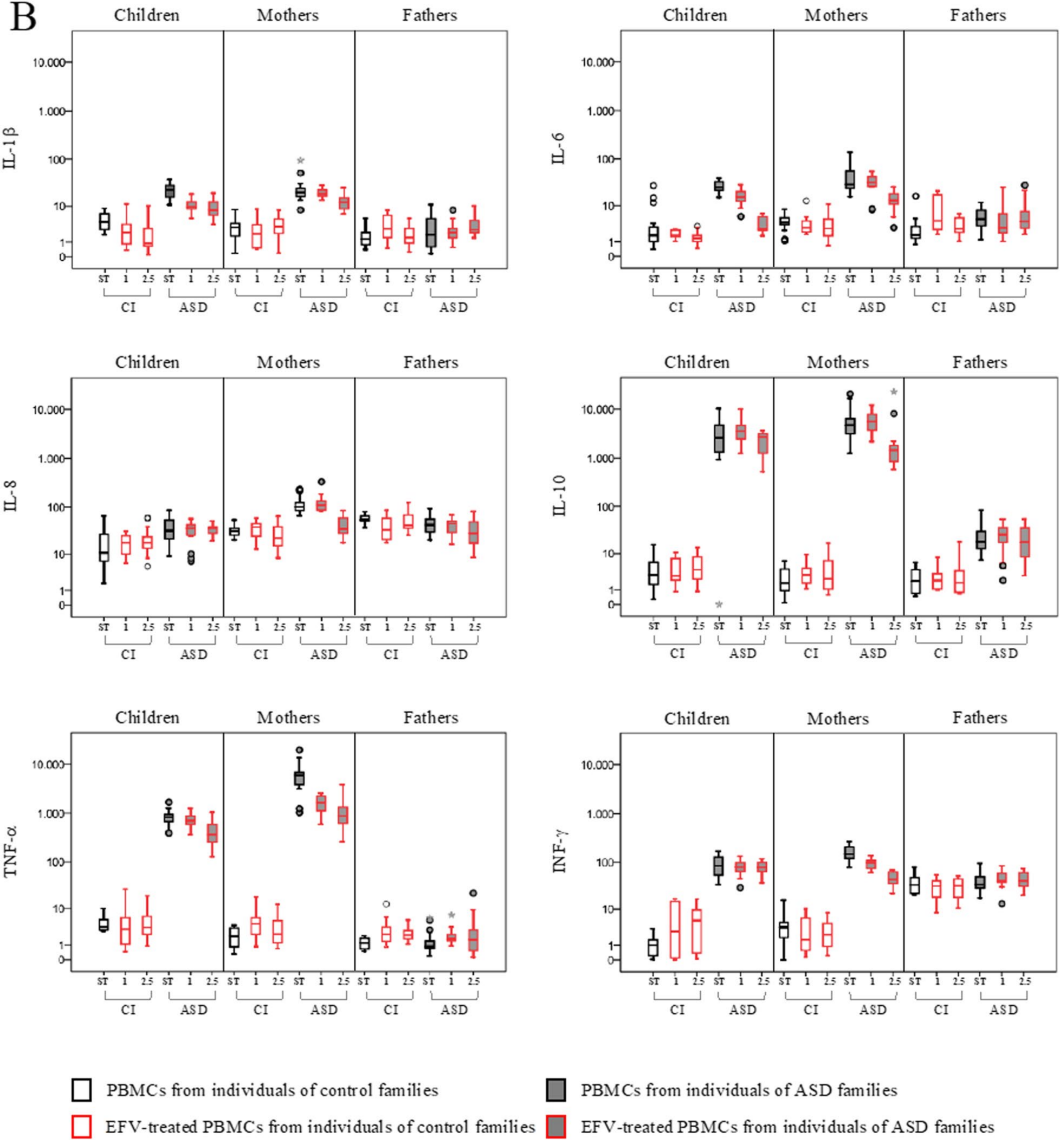
HERVs and cytokines expression in PBMCs from ASD children, their parents and control individuals treated with Efavirenz. HERV transcriptional activity (**A**) and cytokines expression levels (**B**) were analysed in PBMCs treated with Efavirenz (EFV) at a final concentration of 2.5 µM (2.5) and 1 µM (1) by RT-Real time PCR. The results are represented as box plots, depicting mild (black dot), and extreme outliers (asterisk) for each group. Red bordered white boxes = control individuals; Red bordered grey boxes = ASD children and their parents

PBMCs from ASD children and their parents and corresponding controls were also exposed in vitro to Efavirenz (EFV) treatment at the final concentration of 1 and 2.5 µM, and the expression of HERVs and cytokines was evaluated (Fig. 3, Table 3). Median values and IQR of the experimental conditions are reported in Additional file 1: Table S1.

**Table 3:** Non-parametric Friedman test on HERV and cytokines expression in PBMCs exposed in vitro to Efavirenz

		HERV-H	HERV-K	HEMO	HERV-W	pHERV-W	IL-1β	IL-6	IL-8	IL-10	TNF-α	INF-γ
Children	ST *vs *EFV 1 μM	–	0.010	0.023	0.041	–	–	–	–	–	-	0.030
CI	ST *vs *EFV 2.5 μM	–	0.001	–	–	–	–	–	–	–	–	0.027
	EFV 1 μM *vs* EFV2.5 μM	–	–	0.022	0.016	–	–	–	–	–	–	–
Children	ST *vs *EFV 1 μM	0.040	–	0.042	0.016	0.010	0.036	–	–	–	–	–
ASD	ST *vs *EFV 2.5 μM	0.012	0.021	0.046	< 0.001	< 0.001	0.0	–	0.002	–	<0.001	–
	EFV 1 μM *vs* EFV 2.5 μM	–	–	–	–	–	–	–	–	–	0.029	–
Mothers	ST *vs *EFV 1μM	0.022	0.010	0.006	–	–	–	–	–	–	–	–
CI	ST *vs *EFV 2.5 μM	–	0.001	< 0.001	–	–	–	–	–	–	–	–
	EFV 1 μM *vs* EF V2.5 μM	–	–	–	–	–	–	–	–	–	–	–
Mothers	ST *vs *EFV 1 μM	0.005	< 0.001	–	–	–	–	–	–	0.002	–	–
ASD	ST *vs *EFV 2.5 μM	< 0.001	< 0.001	–	–	< 0.001	<0.001	0.002	–	< 0.001	0.005	–
	EFV 1 μM *vs* EFV 2.5 μM	–	-	–	–	< 0.001	0.032	–	–	-	–	–
Fathers	ST *vs *EFV 1 μM	–	-	–	0.010	–	–	–	–	–	–	–
CI	ST *vs *EFV 2.5 μM	0.023	0.026	0.004	0.002	–	–	0.030	–	–	–	–
	EFV 1 μM *vs* EFV 2.5 μM	–	-	–	–	–	–	–	–	–	–	–
Fathers	ST *vs *EFV 1 μM	–	0.002	–	0,021	–	–	–	0.050	–	–	–
ASD	ST *vs *EFV 2.5 μM	–	0.007	–	0.047	–	–	–	0.043	–	–	–
	EFV 1 μM *vs* EFV 2.5 μM	–	-	–	–	–	–	–	–	–	–	–

*HERVs* human endogenous retroviruses, *PBMC*s peripheral blood mononuclear cells*, IL-* Interleukin, *TNF* tumor necrosis factor, *INF* interferon, *CI* control individuals, *ASD* autism spectrum disorder, *T0* basaline, *NS* not stimulated, *ST* stimulated, *VPA* Valproic acid, *EFV* efavirenz

In PBMCs from autistic children, the treatment with EFV significantly reduced the transcriptional activity of HERV-H and HERV-K and conversely significantly induced the expression of HERV-W, regardless of the concentration used. Moreover, treatment with EFV at higher concentration slightly reduced HEMO expression (panel A). In parallel to HERV modulation, EFV treatment also significantly inhibited the expression of IL-6 and TNF-α at the higher concentration (panel B) compared to the untreated condition.

In PBMCs from ASD mothers, EFV exposure significantly reduced HERV-H, HERV-K, HERV-W (at both concentrations) while pHERV-W only at the higher concentration. Concerning cytokines expression, all were significantly reduced at the higher EFV concentration, and TNF-α and INF-γ also at the lower concentration, respect to the untreated condition.

In PBMCs from ASD fathers, the exposure at both concentrations of EFV significantly reduced only HERV-K transcriptional activity, although with values of 2^−∆∆Ct^ < 10.

Among control individuals, exposure to EFV inhibited HERV expression, although variation detected showed values of 2^−∆∆Ct^ < 10. Specifically, EFV significantly reduced the expression of HERV-K in PBMCs from all control individuals, HEMO in PBMCs from mothers, while significantly inducing HERV-W only in fathers.

Furthermore, comparing stimulation response across ASD and CI groups, we found a statistically significant stimulus*diagnostic group interaction effect in children on the reduction of HERV-H (p ≤ 0.004), HERV-K (p ≤ 0.014), IL-6 (p ≤ 0.011) and TNF-α (p ≤ 0.009), and in mothers on the reduction of HERV-H (p = 0.003), HERV-W (p = 0.001), pHERV-W (p < 0.001), IL-1β (p = 0.002), IL-6 (p = 0.001), IL-8 (p = 0.021) and IL-10 (p < 0.001) at the highest concentration analysed, while, only at the lowest concentration of HERV-W (p < 0.001), pHERV-W (p = 0.002) and IL-1β (p = 0.010). As observed for IL2/PHA and VPA, no statically significant results were found in fathers for EFV treatment.

In children at 72 h, we found a statistically significant stimulus*diagnostic group interaction effect on HERV-H (p ≤ 0.004), HERV-K (p ≤ 0.031), IL-1 → (p ≤ 0.001), IL-6 (p ≤ 0.011), TNF-α (p ≤ 0.017) and INF-© (p ≤ 0.050) where the largest interaction effect was observed in IL-2/PHA and VPA.

In mothers at 72 h, we found a statistically significant stimulus*diagnostic group interaction effect on HERV-H (p ≤ 0.049), pHERV-W (p ≤ 0.002), IL-1 → (p ≤ 0.010), IL-6 (p ≤ 0.001), IL-8 (p ≤ 0.021), IL-10 (p ≤ 0.0001) and INF-© (p ≤ 0.018) where the largest interaction effect was observed in VPA and EFV treatment at the higher concentration analyzed.

In fathers no statically significant stimulus*diagnostic group interaction effects were observed.

For the not stimulated condition we didn’t found statically significant stimulus*diagnostic interaction effect on HERVs and cytokines expression in the groups analyzed.

## Discussion

Over the last two decades, scientific research has been focused on the early identification of ASD through signs already evident in the first months of life, suggesting that ASD pathogenesis begins early during development [10, 11]. Furthermore, evidence from preclinical studies suggests that the earlier intervention may yield more improved developmental outcomes [35]. Although a strong genetic component is required, it is now established that several environmental factors increase the risk of autism by acting during pregnancy as additional determinants in genetically susceptible individuals [10]. Such insults could in fact activate the maternal immune response, resulting in an unfavourable developmental environment for the unborn child [11]. Thus, autism could result from the interaction between genetic vulnerability, environmental risk factors, and maternal immune response.

Previously, we demonstrated that both ASD children and their mothers share a molecular trait characterized by high transcriptional activity of HERV-H and HEMO, concomitant with the high expression of TNF-α, IFN-γ and IL-10 in fresh PBMCs, suggesting a possible mother–child association in ASD. Hence, we propose HERVs as contributing factors to the etiopathogenesis of autism, representing the link between genetic susceptibility and environmental risk factors [12, 32].

Herein, we investigated whether abnormal expression of HERVs and cytokines found in PBMCs from autistic children and their parents can be modulated by exposing PBMCs to stimulating factors, such as IL-2 and PHA, or drugs, such as the antiepileptic drug valproic acid and the antiretroviral drug efavirenz. Notable, the results demonstrated that PBMCs from ASD children and their mothers share intrinsic responsiveness to microenvironmental stimuli. Indeed, the in vitro exposure to IL-2 and PHA significantly increased HERV-H, HERV-K and HEMO in ASD children and pHERV-W in their mothers. It has been hypnotized that HERVs could be involved in ASD pathogenesis due to their intrinsic responsiveness to microenvironmental stress and external stimuli, such as nutrients, hormones, cytokines and pathogens, likely via epigenetic mechanisms [25, 36]. The epigenetic regulation is indeed essential during embryonic development leading to cell commitment and tissues specification [37], when a global remodeling occurs, and any alteration could impact neurodevelopment and cognitive function [38]. In this context, HERV dysregulation could potentially influence central nervous system development. Accordingly, non-coding RNA (ncRNAs) expressed by the HERV-H group and the recruitment of specific cellular transcriptional factors on HERV-H Long Terminal Repeats (LTRs) are involved in the conservation of stem cell identity during embryogenesis [39, 40]. HERV-K is also required in embryogenesis during the preimplantation phase and in maintaining the pluripotent state of embryonic cells [41]. Finally, it is interesting to note that HEMO, an ancestral env gene in humans, has also been found expressed in embryos, already in the early stages of development and all subsequent periods of differentiation [31]. On the other side, the pHERV-W, initially referred as the multiple sclerosis-associated retrovirus (MSRV), encodes an immunopathogenic and neurotoxic envelope protein (HERV-W ENV), and has been associated with human inflammatory and autoimmune diseases and recently with COVID-19 [26, 42]. In COVID-19 patients, elevated HERV-W ENV protein expression has been found associated with certain pathogenic features of the disease, peculiarly in more severe cases. Moreover, in vitro exposure of PBMCs from healthy donors to SARS-CoV-2 Spike protein induced an early expression of HERV-W ENV, and ahead of the induction of IL-6, suggesting early HERV activation consistent with its potential role in the inflammation process related to infectious diseases [42].

Furthermore, in the present study, we also observed that cytokine expression was modulated in parallel to HERVs, since in vitro stimulation induced the expression of IL-1β, IL-6, TNF-α, IFN-γ in PBMCs from ASD children and their mothers and IL-8 and IL-10 only in ASD mothers. Accordingly, growing evidence suggests a link between immune system dysregulation and autism, with immune abnormalities described in individuals with ASD as well as their family members [32, 43, 44]. In ASD children high levels of IFN-γ were found in the brain, and IL-6 and TNF-α in the blood and cerebrospinal fluid [45, 46]. An elevated expression of several cytokines, like IFN-γ, IL-1β and IL-8 was also observed in the serum of pregnant women who had given birth to children later diagnosed autistic [47, 48]. Interestingly, IL-6 can contribute to neurodevelopmental disorders like autism and schizophrenia, as suggested by the observation that the administration of IL-6 to pregnant dams mimics the effects of maternal immune activation on the upregulation of genes implicated in autism and schizophrenia in foetal brain tissue [49]. Moreover, IL-6 can regulate cognitive-associated genes via promotors localized in the LTRs of the MER41 family of primate-specific HERVs, underling the connection among immune, LTR-mediated gene expression and cognitive pathways in human disabilities [50]. High levels of IL-6 and IL-1β have been also associated with more severe behavioural deficits in children with autism [45], while high levels of the anti-inflammatory marker IL-10 have been found in individuals with mild autism-related behavioural impairments [51]. Of note, IL-10 is an important crosstalk mediator between maternal immunity and foetal growth, and recently it has been proposed that maternal genetics may influence the foetal neurodevelopment and therefore also the autistic phenotype in offspring through the alteration of IL-10-mediated maternal-foetal immunosuppression [52]. It is worth mentioning that ASD children and their mothers share intrinsic responsiveness of PBMCs to microenvironmental changes in expressing HERVs and pro-inflammatory cytokines, whereas no association was found with fathers or in control families. The common expression profile in ASD children and their mothers, and the discrepancy with fathers, support the hypothesis of maternal imprinting as a contributing factor in increasing susceptibility to neurodevelopmental disorders and also suggests that the interaction between HERV activity and inflammation may play a pivotal role in the etiopathogenesis of ASD [12, 32]. Maternal imprinting could involve mitochondrial activity, as these organelles are inherited exclusively via maternal lineage. Mitochondrial dysfunctions are described in autism [53] and it’s well established the that mitochondria play an important role in modulating innate immune response [54]. Nevertheless, also father-associated genetic factors could contribute to the development of the disorder, through pathways that have not yet been clarified [55, 56].

HERVs can shape innate immune response by mechanisms, including the regulation of the expression of neighboring genes and the stimulation of pattern recognition receptors (PRRs). The up-regulation of HERV transcription can lead to the release of HERV-derived pathogen-associated molecular patterns (PAMPs), that evoke the production of pro-inflammatory effectors [57]. Since HERVs are physiologically expressed in humans [58] or can be activated by microenvironmental changes, they could provide continuous triggers to the host innate immune sensors. On the other side, the inflammatory effectors could in turn further increase HERV activity.

Herein, we demonstrated that also the in vitro exposure to VPA modified the expression of different HERVs and cytokines. It’s well-established that VPA administration in pregnant women has been associated with an increased risk of autism in the offspring [59, 60]. VPA could act directly, interfering with the neurotransmission process, or indirectly via epigenetic regulation of genes implicated in immune system and brain function and development [61–63]. Here we observed that VPA treatment modulated the expression of HERV-H, HERV-W, pHERV-W, IL-8 and TNF-α in PBMCs from ASD children and HERV-H, HERV-K, pHERV-W, IL-1β, IL-6, IL-10 and TNF-α in PBMCs from their mothers. The concomitant modulation of HERVs and cytokines is in line with our previous findings in preclinical studies in which, beginning from intrauterine life and up to adulthood, high expression levels of ERVs and cytokines were found in mice prenatally exposed to valproic acid [33]. Interestingly, the altered ERV expression was transgenerationally transmitted via maternal lineage, probably through epigenetic mechanisms, in parallel to the autistic-like phenotype [34]. Moreover, the exposure of pregnant rodents to infectious agents (e.g., influenza virus, Escherichia coli) or to a synthetic analogue of viral double-stranded RNA [polyinosinic–polycytidylic acid (Poly (I:C)], leads to the maternal immune activation and the production of proinflammatory cytokines [64–66]. Cytokines can cross the placenta, or be produced in loco, with a consequent gene dysregulation in the foetus, affecting brain function and development and leading to a permanent dysregulation of the immune system in the offspring [67, 68].

We also investigated whether exposure to an antiretroviral drug can specifically restore the abnormal HERV activity found in PBMCs from ASD children and their mothers. The use of antiretroviral drugs to inhibit HERV expression has been already proven in vitro and proposed in the setting of combined therapy in cancer and neurological conditions in which HERVs have been implicated [69–73]. Indeed, EFV has already been shown to inhibit the endogenous reverse transcriptase activity in leukemic cells and several human cell lines [70–72, 74]*,* and we previously demonstrated that the antiretroviral drugs azidothymidine and EFV can inhibit the expression of HERV-K and HERV-H in cancer cells under unfavorable culture conditions [69, 75]. Herein we found that EFV modulates HERV activity in PBMCs from ASD children and their mothers, restoring values similar to those of the corresponding controls. Indeed, EFV treatment reduced HERV-H and HERV-K expression in PBMCs from ASD children and their mothers and HERV-W and pHERV-W only in ASD mothers, while, conversely, increased HERV-W activity in ASD children. The reduction of pHERV-W observed in ASD mothers is in line with the results reported by Morandi et al. in lymphoblastoid cell lines [73], and with the hypothesis that antiretroviral therapy for HIV could potentially limit the progression of multiple sclerosis [76–78]. Notable, EFV treatment in PBMCs from ASD children also reduced the expression of the pro-inflammatory cytokines IL-1→, IL-6 and TNF-α, maintaining at high levels the expression of regulatory cytokine IL-10, while in PBMCs from ASD mothers reduced the expression of all the cytokines analysed, although maintaining high levels of IL-10 and IFN©. Thus, EFV specifically restored the expression of certain HERVs with a concomitant modulation of cytokines, in particular lowering the proinflammatory while maintaining high the regulatory ones, underlining the close interplay between HERV activity and inflammation. Another mechanism by which HERVs could contribute to pathogenic mechanisms involved in ASD concerns the possibility that HERV transcripts may act with RNA-mediated toxicity. RNA-mediated mechanisms of disease pathogenesis have been recognized implicated in the context of neurological disorders, such as myoclonic epilepsy, myotonic dystrophy, Fragile X syndrome, Huntington’s disease-like 2 and amyotrophic lateral sclerosis [79–83]. Intriguingly, a recent paper indicates a potential new function of HERV-K transcripts in neurodegeneration, via trigger TLR7 and TLR8 signaling in a mouse model of Alzheimer’s disease [84].

The main limitation of our study is the small number of the individuals enrolled; therefore, our findings need to be replicated in larger studies. Moreover, a different response of lymphocytes to in vitro stimulation in expressing HERVs and cytokines depending on the subjects’ age cannot be excluded, but no correlations emerged in our cohort (data not shown) and also this could be related to the relative small sample size. In addition, this is an in vitro study on PBMCs, given the inability of accessing human neurons. A protocol for transdifferentiating human circulating monocytes into neuron-like cells, expressing several genes and proteins associated with neuronal structure, could circumvent this obstacle [85].

## Conclusions

HERVs have been proposed as contributing factors in autism due to their role in human embryogenesis, their intrinsic responsiveness to stimuli, and their interaction with the immune system.

Here we show that HERVs and cytokines expression can be modulated in PBMCs from autistic children and their mothers by in vitro with different stimuli, and interestingly the antiretroviral drug Efavirenz restores the expression of specific HERV families to values similar to those of the controls, also reducing the expression of proinflammatory cytokines but keeping the regulatory ones high. Although we are far from demonstrating a causal relationship between deregulation of HERV expression, immune alteration, and autism, our findings could open new perspectives to study the pathophysiology of HERVs and biological mechanisms underlying neurodevelopmental derailment. Moreover, through the use of preclinical models of autism it could be elucidated whether the administration of antiretroviral drugs during pregnancy can prevent or improve the altered behavioral phenotype in offspring.

## Materials and methods

### Demographic characteristics of ASD children and control individuals

The study was carried out on a subgroup of the cohort of ASD and control individuals (CI) families used in our previous study [32]. Specifically, here we analysed PBMCs obtained from a total of 112 individuals belonging to 35 families, of which 21 with ASD children and 14 with typical-developing children; five control families were composed of two children, one of three children, and the remaining of one child. In Table 4 the demographic information of individuals included in the present study is reported. No statistical differences were found in the comparison of the median age among the children, mothers and fathers’ groups, and for sex ratio between the children groups.

**Table 4:** Demographic information of individuals included in the study

	ASD	Control individuals
Numbers of families	21	14
Children (number)	21	21
Male	18	18
Female	3	3
Ratio male/female	6	6
Median age (range)	4.8(2–10)	5(2–17)
Mothers (number)	21	14
Median age (range)	35.3(31–42)	36.5(26–43)
Fathers (number)	21	14
Median age (range)	40.67(29–47)	43.5(29–47)

The families including ASD individuals were recruited at the Child Psychiatry and Neurology Unit of “Tor Vergata” University (Rome, Italy); children with ASD were diagnosed according to the Diagnostic and Statistical Manual of Mental Disorders-IV, text revision (DSM-IV-TR) as Autistic Disorder (AD; 21/21) and confirmed by the DSM-5 criteria [2]. All ASD children performed a clinical evaluation by Autism Diagnostic Observation Schedule (ADOS-2), including a specific index of symptoms severity calculated with Calibrate Severity Score (CSS = 4.815; range 1–10). ASD children with known infectious, metabolic or genetic diseases, chromosomal abnormalities, seizures, identifiable neurological syndromes, or focal signs were excluded from the study.

Age- and sex-matched healthy volunteers were recruited among the employees of the Medicine Faculty of “Tor Vergata” University and the Child Neurology and Psychiatry Unit of “Tor Vergata” Hospital (Rome, Italy) and enrolled together with their typical developing children who attended outpatient facilities for routine examinations. None of them reported neurological or psychiatric disorders or the presence of ongoing infections in their medical history. All enrolled individuals were not taking any medications at the sampling time.

### Peripheral blood mononuclear cells isolation and culture conditions

PBMCs were isolated from blood samples, after dilution 1:4 with phosphate-buffered saline (PBS) (Sigma-Aldrich, MO, USA), by density gradient centrifugation (Lympholyte-H, Merck Darmstadt, Germany). After washing with PBS, PBMCs were counted and collected immediately (condition named basal level, T_0_) or seeded in 24-well tissue culture plate in RPMI 1640 medium supplemented with 12% (v/v) heat-inactivated fetal bovine serum (FBS), L-glutamine (2 mM), penicillin (50 U/ml), streptomycin (50 U/ml) (all from Sigma-Aldrich, MO, USA). For the purpose of the study, PBMCs were maintained in culture without any stimulations (hereinafter NS) or in presence of Interleukin-2 (IL-2, 20U/ml) and Phytohaemagglutinin (PHA, 2 μg/ml), (hereinafter ST) for 72 h. Moreover, to evaluate the effect of Efavirenz (kindly provided by Corrado Spadafora and named hereinafter EFV) and Valproic acid (Sigma-Aldrich, MO, USA; hereinafter VPA), the drugs were added at the time of setting up the culture, simultaneously with IL-2 (20U / ml) and PHA, (2 μg/ml), and cells were maintained at 37 °C in a humidified 5% CO_2_ atmosphere. After 72 h of treatment, PBMCs were collected, washed twice with PBS, recovered by 8 min centrifugation at 600 g at 4 °C, and stored at -80 °C.

### RNA extraction and RT-real time PCR

Total RNA was extracted from PBMCs using the NucleoSpin RNA kit according to the manufacturer’s instructions (Macherey–Nagel, Dueren, Germany). Two hundred fifty nanograms of DNase-treated RNA were reverse-transcribed into cDNA using Improm-II Reverse Transcription System (Promega, Fitchburg, WI, USA) according to the manufacturer’s protocol, in a total volume of 20 µl. The transcriptional levels of selected HERVs and cytokines were assessed by Real-time PCR in CFX96 Real-Time System (Bio-Rad), using SYBR Green chemistry (iTaq Universal SYBR green Supermix, Biorad). Specific pairs of primers for env of HERV-H, HERV-K, HERV-W [29], HEMO [31] and pHERV-W [42], and cytokines expression including IL-1β, IL-6, IL-8, IL-10, TNF-α, IFN-γ [32] were used.

To set up the Real-Time PCR a serial dilution (tenfold) was done to calculate efficiencies and correlation coefficient. The amplification efficiency was calculated by the formula [efficiency = 10(− 1/slope)] and all the primer pairs showed an efficiency ranging from 0.95 to 0.97. To verify the specificity of the primers and to exclude any false positives, DNA sequencing of PCR samples from individuals belonging to ASD and control families, was performed. Real-Time PCR included 2.0 µl of 1:10 diluted cDNA, 10 µl of SYBR green Supermix, and specific primers ranging from 100 to 200 nM, in a total volume of 20 µl, and was conducted for 1 cycle at 95◦C for 5 min and then for 45 cycles of 95◦C for 10 s and 60◦C for 15 s. Each sample was analyzed in triplicate and to check out any possible contamination, a negative control was included in each experiment. The housekeeping gene β-glucuronidase (GUSB) [29] was used to normalize the results. Each experiment was completed with a melting curve analysis to confirm the specificity of amplification and the lack of non-specific products and primer dimers. Quantification was performed using the threshold cycle (Ct) comparative method and the relative expression was calculated as follows 2^−[ΔCt(sample)− ΔCt(calibrator)^ = 2^−ΔΔCt^ where ΔCt (sample) = [Ct (target gene)-Ct (housekeeping gene)] and ΔCt (calibrator) was the mean of ΔCT of all the samples from typical developing individuals.

### Statistical analysis

Statistical analysis of group-wise expression levels was performed through the non-parametric Friedman test. In order to compare stimulation response across groups (ASD and CI) while taking confounds into account, for each stimulus we employed linear mixed models (with each biomarker as the dependent variable) which included timepoint as repeated within-subject factor (baseline vs 72 h), a between-subject factor (diagnostic group), a timepoint*diagnostic group interaction term, as well as age, sex, and kinship (coded as family membership) as covariates of no interest. All mixed models employed unstructured estimate of the covariance matrix, i.e. all covariance elements were estimated directly from data. The analysis was repeated separately in children mothers, and fathers. Data analyses were performed using SPSS statistical software system (version 24.0 for Windows, USA). Statistically significant comparisons were considered when p < 0.050.

## Supplementary Information

**Additional file 1: Table S1**. Median values and interquartile range (IQR) of HERVs and cytokines expression in PBMCs from autistic children, their parents and corresponding healthy controls. HERVs: human endogenous retroviruses; PBMCs: peripheral blood mononuclear cells; CI: control individuals; ASD: autism spectrum disorder; T0: basal level, NS: not stimulated; ST: stimulated; VPA: Valproic acid; EFV: Efavirenz.

## Data Availability

The datasets analysed during the current study are available from the corresponding author on reasonable request.

## Abbreviations

ADHD: Attention deficit/ hyperactivity disorder
ADOS: Autism diagnostic observation schedule
ASD: Autism spectrum disorder
T0: Basaline;
CI: Control individuals
CSS: Calibrate severity score
DSM-IV-TR: Diagnostic and statistical manual of mental disorders, 4th edition, text revision
EFV: Efavirenz
FBS: Heat-inactivated fetal bovine serum
GUSB: β-Glucuronidase
HEMO: Human endogenous MER34 (medium-reiteration-frequency-family-34) ORF
HERVs: Human endogenous retroviruses
IL: Interleukin
INF-γ: Interferon gamma
IQR: Interquartile range
LTRs: Long terminal repeats
MSRV: Multiple sclerosis-associated retroviruses
ncRNAs: Non-coding RNA
NS: Without any stimulation
PAMPs: Derived pathogen-associated molecular patterns
PBMC: Peripheral blood mononuclear cell
PBS: Phosphate-buffered saline
PHA: Phytohaemagglutinin
Poly(I:C): Polyinosinic–polycytidylic acid
PRRs: Pattern recognition receptors
ST: In presence of IL-2 and PHA; TNF-α, tumor necrosis factor alpha
VPA: Valproic acid

## References

[1] Lord C, Elsabbagh M, Baird G, Veenstra-Vanderweele J (2018). Autism spectrum disorder. Lancet.

[2] American Psychiatric Association (2013). Diagnostic and statistical manual of mental disorders.

[3] Emberti Gialloreti L, Curatolo P (2018). Autism spectrum disorder: why do we know so little?. Front Neurol.

[4] Sandin S, Lichtenstein P, Kuja-Halkola R, Hultman C, Larsson H, Reichenberg A (2017). The heritability of autism spectrum disorder. JAMA.

[5] Grove J, Ripke S, Als TD, Mattheisen M, Walters RK, Won H, Pallesen J (2019). Identification of common genetic risk variants for autism spectrum disorder. Nat Genet.

[6] Rosenberg RE, Law JK, Yenokyan G, McGready J, Kaufmann WE, Law PA (2009). Characteristics and concordance of autism spectrum disorders among 277 twin pairs. Arch Pediatr Adolesc Med.

[7] Hallmayer J, Cleveland S, Torres A, Phillips J, Cohen B, Torigoe T (2011). Genetic heritability and shared environmental factors among twin pairs with autism. Arch Gen Psychiatry.

[8] Lee GA, Lin YK, Lai JH, Lo YC, Yang YSH, Ye SY (2021). Maternal immune activation causes social behavior deficits and hypomyelination in male rat offspring with an autism-like microbiota profile. Brain Sci.

[9] Abuaish S, Al-Otaibi NM, Abujamel TS, Alzahrani SA, Alotaibi SM, AlShawakir YA (2021). Fecal transplant and bifidobacterium treatments modulate gut clostridium bacteria and rescue social impairment and hippocampal BDNF expression in a rodent model of autism. Brain Sci.

[10] Heyer DB, Meredith RM (2017). Environmental toxicology: sensitive periods of development and neurodevelopmental disorders. Neurotoxicology.

[11] Estes ML, McAllister AK (2016). Maternal immune activation: implications for neuropsychiatric disorders. Science.

[12] Balestrieri E, Matteucci C, Cipriani C, Grelli S, Ricceri L, Calamandrei G (2019). Endogenous retroviruses activity as a molecular signature of neurodevelopmental disorders. Int J Mol Sci.

[13] Belshaw R, Pereira V, Katzourakis A, Talbot G, Paces J, Burt A (2004). Long-term reinfection of the human genome by endogenous retroviruses. Proc Natl Acad Sci U S A.

[14] Bannert N, Kurth R (2006). The evolutionary dynamics of human endogenous retroviral families. Annu Rev Genomics Hum Genet.

[15] Bock M, Stoye JP (2000). Endogenous retroviruses and the human germline. Curr Opin Genet Dev.

[16] Lander ES, Linton LM, Birren B, Nusbaum C, Zody MC, Baldwin J (2001). Initial sequencing and analysis of the human genome. Nature.

[17] Feschotte C, Gilbert C (2012). Endogenous viruses: insights into viral evolution and impact on host biology. Nat Rev Genet.

[18] Dewannieux M, Heidmann T (2013). Endogenous retroviruses: acquisition, amplification and taming of genome invaders. Curr Opin Virol.

[19] Tristem M (2000). Identification and characterization of novel human endogenous retrovirus families by phylogenetic screening of the human genome mapping project database. J Virol.

[20] Bénit L, Dessen P, Heidmann T (2001). Identification, phylogeny, and evolution of retroviral elements based on their envelope genes. J Virol.

[21] Thomas J, Perron H, Feschotte C (2018). Variation in proviral content among human genomes mediated by LTR recombination. Mob DNA.

[22] Belshaw R, Katzourakis A, Paces J, Burt A, Tristem M (2005). High copy number in human endogenous retrovirus families is associated with copying mechanisms in addition to reinfection. Mol Biol Evol.

[23] Wildschutte J, Williams Z, Montesion M, Subramanian R, Kidd J, Coffin J (2016). Discovery of unfixed endogenous retrovirus insertions in diverse human populations. Proc Natl Acad Sci.

[24] Frank JA, Feschotte C (2017). Co-option of endogenous viral sequences for host cell function. Curr Opin Virol.

[25] Matteucci C, Balestrieri E, Argaw-Denboba A, Sinibaldi-Vallebona P (2018). Human endogenous retroviruses role in cancer cell stemness. Semin Cancer Biol.

[26] Küry P, Nath A, Créange A, Dolei A, Marche P, Gold J (2018). Human endogenous retroviruses in neurological diseases. Trends Mol Med.

[27] Leboyer M, Tamouza R, Charron D, Faucard R, Perron H (2013). Human Endogenous retrovirus type W (HERV-W) in schizophrenia: a new avenue of research at the gene-environment interface. World J Biol Psychiatry.

[28] Levet S, Charvet B, Bertin A, Deschaumes A, Perron H, Hober D (2019). Human endogenous retroviruses and type 1 diabetes. Curr Diab Rep.

[29] Balestrieri E, Arpino C, Matteucci C, Sorrentino R, Pica F, Alessandrelli R (2012). HERVs expression in autism spectrum disorders. PLoS ONE.

[30] Balestrieri E, Pitzianti M, Matteucci C, D’Agati E, Sorrentino R, Baratta A (2014). Human endogenous retroviruses and ADHD. World J Biol Psychiatry.

[31] Heidmann O, Béguin A, Paternina J, Berthier R, Deloger M, Bawa O (2017). HEMO, an ancestral endogenous retroviral envelope protein shed in the blood of pregnant women and expressed in pluripotent stem cells and tumors. Proc Natl Acad Sci U S A.

[32] Balestrieri E, Cipriani C, Matteucci C, Benvenuto A, Coniglio A, Argaw-Denboba A (2019). Children with autism spectrum disorder and their mothers share abnormal expression of selected endogenous retroviruses families and cytokines. Front Immunol.

[33] Cipriani C, Ricceri L, Matteucci C, De Felice A, Tartaglione AM, Argaw-Denboba A (2018). High expression of endogenous retroviruses from intrauterine life to adulthood in two mouse models of autism spectrum disorders. Sci Rep.

[34] Tartaglione AM, Cipriani C, Chiarotti F, Perrone B, Balestrieri E, Matteucci C (2019). early behavioral alterations and increased expression of endogenous retroviruses are inherited across generations in mice prenatally exposed to valproic acid. Mol Neurobiol.

[35] Grzadzinski R, Amso D, Landa R, Watson L, Guralnick M, Zwaigenbaum L (2021). Pre-symptomatic intervention for autism spectrum disorder (ASD): defining a research agenda. J Neurodevelop Disord.

[36] Perron H, Lang A (2010). The human endogenous retrovirus link between genes and environment in multiple sclerosis and in multifactorial diseases associating neuroinflammation. Clin Rev Allergy Immunol.

[37] Gropman AL, Batshaw ML (2010). Epigenetics, copy number variation, and other molecular mechanisms underlying neurodevelopmental disabilities: new insights and diagnostic approaches. J Dev Behav Pediatr.

[38] LaSalle JM (2013). Epigenomic strategies at the interface of genetic and environmental risk factors for autism. J Hum Genet.

[39] Wang J, Xie G, Singh M, Ghanbarian AT, Raskó T, Szvetnik A (2014). Primate-specific endogenous retrovirus-driven transcription defines naive-like stem cells. Nature.

[40] Glinsky GV (2015). Transposable elements and DNA methylation create in embryonic stem cells human-specific regulatory sequences associated with distal enhancers and noncoding RNAs. Genome Biol Evol.

[41] Grow EJ, Flynn RA, Chavez SL, Bayless NL, Wossidlo M, Wesche DJ (2015). Intrinsic retroviral reactivation in human preimplantation embryos and pluripotent cells. Nature.

[42] Balestrieri E, Minutolo A, Petrone V, Fanelli M, Iannetta M, Malagnino V (2021). Evidence of the pathogenic HERV-W envelope expression in T lymphocytes in association with the respiratory outcome of COVID-19 patients. EBioMedicine.

[43] Goines P, Van de Water J (2010). The immune systems role in the biology of autism. Curr Opin Neurol.

[44] Gottfried AW, Schlackman J, Gottfried AE, Boutin-Martinez AS (2015). Parental provision of early literacy environment as related to reading and educational outcomes across the academic lifespan. Parenting.

[45] Masi A, Breen EJ, Alvares GA, Glozier N, Hickie IB, Hunt A (2017). Cytokine levels and associations with symptom severity in male and female children with autism spectrum disorder. Mol Autism.

[46] Goines PE, Ashwood P (2013). Cytokine dysregulation in autism spectrum disorders (ASD): possible role of the environment. Neurotoxicol Teratol.

[47] Ashwood P, Krakowiak P, Hertz-Picciotto I, Hansen R, Pessah I, Van de Water J (2011). Elevated plasma cytokines in autism spectrum disorders provide evidence of immune dysfunction and are associated with impaired behavioral outcome. Brain Behav Immun.

[48] Masi A, Quintana DS, Glozier N, Lloyd AR, Hickie IB, Guastella AJ (2015). Cytokine aberrations in autism spectrum disorder: a systematic review and meta-analysis. Mol Psychiatry.

[49] Garbett KA, Hsiao EY, Kálmán S, Patterson PH, Mirnics K (2012). Effects of maternal immune activation on gene expression patterns in the fetal brain. Transl Psychiatry.

[50] Nataf S, Uriagereka J, Benitez-Burraco A (2019). The promoter regions of intellectual disability-associated genes are uniquely enriched in LTR sequences of the MER41 primate-specific endogenous retrovirus: an evolutionary connection between immunity and cognition. Front Genet.

[51] Ross HE, Guo Y, Coleman K, Ousley O, Miller AH (2013). Association of IL-12p70 and IL-6:IL-10 ratio with autism-related behaviors in 22q11.2 deletion syndrome: a preliminary report. Brain Behav Immun.

[52] Jaini R, Wolf MR, Yu Q, King AT, Frazier TW, Eng C (2021). Maternal genetics influences fetal neurodevelopment and postnatal autism spectrum disorder-like phenotype by modulating in-utero immunosuppression. Transl Psychiatry.

[53] Frye RE (2020). Mitochondrial dysfunction in autism spectrum disorder: unique abnormalities and targeted treatments. Semin Pediatr Neurol.

[54] West AP, Shadel GS, Ghosh S (2011). Mitochondria in innate immune responses. Nat Rev Immunol.

[55] Cioppi F, Casamonti E, Krausz C (2019). Age-dependent de novo mutations during spermatogenesis and their consequences. Adv Exp Med Biol.

[56] Breuss MW, Antaki D, George RD, Kleiber M, James KN, Ball LL (2020). Autism risk in offspring can be assessed through quantification of male sperm mosaicism. Nat Med.

[57] Grandi N, Tramontano E (2018). Human endogenous retroviruses are ancient acquired elements still shaping innate immune responses. Front Immunol.

[58] Balestrieri E, Pica F, Matteucci C, Zenobi R, Sorrentino R, Argaw-Denboba A (2015). Transcriptional activity of human endogenous retroviruses in human peripheral blood mononuclear cells. Biomed Res Int.

[59] Christensen J, Grønborg TK, Sørensen MJ, Schendel D, Parner ET, Pedersen LH (2013). Prenatal valproate exposure and risk of autism spectrum disorders and childhood autism. JAMA.

[60] Bromley RL, Mawer GE, Briggs M, Cheyne C, Clayton-Smith J, García-Fiñana M (2013). The prevalence of neurodevelopmental disorders in children prenatally exposed to antiepileptic drugs. J Neurol Neurosurg Psychiatry.

[61] Manent J-B, Jorquera I, Mazzucchelli I, Depaulis A, Perucca E, Ben-Ari Y (2007). Fetal exposure to GABA-acting antiepileptic drugs generates hippocampal and cortical dysplasias. Epilepsia.

[62] Fukuchi M, Nii T, Ishimaru N, Minamino A, Hara D, Takasaki I (2009). Valproic acid induces up- or down-regulation of gene expression responsible for the neuronal excitation and inhibition in rat cortical neurons through its epigenetic actions. Neurosci Res.

[63] Kawanai T, Ago Y, Watanabe R, Inoue A, Taruta A, Onaka Y (2016). Prenatal exposure to histone deacetylase inhibitors affects gene expression of autism-related molecules and delays neuronal maturation. Neurochem Res.

[64] Gilmore JH, Jarskog LF, Vadlamudi S (2005). Maternal poly I: C exposure during pregnancy regulates TNF alpha, BDNF, and NGF expression in neonatal brain and the maternal-fetal unit of the rat. J Neuroimmunol.

[65] Urakubo A, Jarskog LF, Lieberman JA, Gilmore JH (2001). Prenatal exposure to maternal infection alters cytokine expression in the placenta, amniotic fluid, and fetal brain. Schizophr Res.

[66] Zuckerman L, Weiner I (2005). Maternal immune activation leads to behavioral and pharmacological changes in the adult offspring. J Psychiatr Res.

[67] Kempuraj D, Thangavel R, Selvakumar GP, Zaheer S, Ahmed ME, Raikwar SP (2017). Brain and peripheral atypical inflammatory mediators potentiate neuroinflammation and neurodegeneration. Front Cell Neurosci.

[68] Abdallah MW, Larsen N, Grove J, Nørgaard-Pedersen B, Thorsen P, Mortensen EL (2013). Amniotic fluid inflammatory cytokines: potential markers of immunologic dysfunction in autism spectrum disorders. World J Biol Psychiatry.

[69] Argaw-Denboba A, Balestrieri E, Serafino A, Cipriani C, Bucci I, Sorrentino R (2017). HERV-K activation is strictly required to sustain cd133+ melanoma cells with stemness features. J Exp Clin Cancer Res.

[70] Sinibaldi-Vallebona P, Matteucci C, Spadafora C (2011). Retrotransposon-encoded reverse transcriptase in the genesis, progression and cellular plasticity of human cancer. Cancers.

[71] Tyagi R, Li W, Parades D, Bianchet MA, Nath A (2017). Inhibition of human endogenous retrovirus-k by antiretroviral drugs. Retrovirology.

[72] Contreras-Galindo R, Dube D, Fujinaga K, Kaplan MH, Markovitz DM (2017). Susceptibility of human endogenous retrovirus type k to reverse transcriptase inhibitors. J Virol.

[73] Morandi E, Tanasescu R, Tarlinton RE, Constantin-Teodosiu D, Gran B (2019). Do antiretroviral drugs protect from multiple sclerosis by inhibiting expression of ms-associated retrovirus?. Front Immunol.

[74] Mangiacasale R, Pittoggi C, Sciamanna I, Careddu A, Mattei E, Lorenzini R (2003). Exposure of normal and transformed cells to nevirapine, a reverse transcriptase inhibitor, reduces cell growth and promotes differentiation. Oncogene.

[75] Giovinazzo A, Balestrieri E, Petrone V, Argaw-Denboba A, Cipriani C, Miele MT (2019). The concomitant expression of human endogenous retroviruses and embryonic genes in cancer cells under microenvironmental changes is a potential target for antiretroviral drugs. Cancer Microenviron.

[76] Gold J, Goldacre R, Maruszak H, Giovannoni G, Yeates D, Goldacre M (2015). HIV and lower risk of multiple sclerosis: beginning to unravel a mystery using a record-linked database study. J Neurol Neurosurg Psychiatry.

[77] Chalkley J, Berger JR (2014). Multiple sclerosis remission following antiretroviral therapy in an hiv-infected man. J Neurovirol.

[78] Mainardi I, Ferrò MT, Gastaldi M, Franciotta D, Cinque P (2020). Acquisition of human immunodeficiency virus infection in a patient with multiple sclerosis: could these conditions positively influence each other’s course?. J Neurovirol.

[79] Corbett MA, Kroes T, Veneziano L, Bennett MF, Florian R, Schneider AL (2019). Intronic ATTTC repeat expansions in STARD7 in familial adult myoclonic epilepsy linked to chromosome 2. Nat Commun.

[80] Mankodi A, Lin X, Blaxall BC, Swanson MS, Thornton CA (2005). Nuclear RNA foci in the heart in myotonic dystrophy. Circ Res.

[81] Greco CM, Berman RF, Martin RM, Tassone F, Schwartz PH, Chang A (2006). Neuropathology of fragile X-associated tremor/ataxia syndrome (FXTAS). Brain.

[82] Rudnicki DD, Holmes SE, Lin MW, Thornton CA, Ross CA, Margolis RL (2007). Huntington’s disease-like 2 is associated with CUG repeat-containing RNA foci. Ann Neurol.

[83] Donnelly CJ, Zhang P-W, Pham JT, Haeusler AR, Heusler AR, Mistry NA (2013). RNA toxicity from the ALS/FTD C9ORF72 expansion is mitigated by antisense intervention. Neuron.

[84] Dembny P, Newman AG, Singh M, Hinz M, Szczepek M, Krüger C (2020). Human endogenous retrovirus HERV-K(HML-2) RNA causes neurodegeneration through toll-like receptors. JCI Insight.

[85] Bellon A, Hasoglu T, Peterson M, Gao K, Chen M, Blandin E (2021). Optimization of neurite tracing and further characterization of human monocyte-derived-neuronal-like cells. Brain Sci.

